# The influence of developmental diet on reproduction and metabolism in *Drosophila*

**DOI:** 10.1186/s12862-020-01663-y

**Published:** 2020-07-29

**Authors:** Peter Klepsatel, Diana Knoblochová, Thirnahalli Nagaraj Girish, Heinrich Dircksen, Martina Gáliková

**Affiliations:** 1 Institute of Zoology, Slovak Academy of Sciences, Dúbravská cesta 9, 845 06 Bratislava, Slovakia; 2 Department of Genetics, Faculty of Natural Sciences, Comenius University, Ilkovičova 6, Mlynská dolina, 84215 Bratislava, Slovakia; 3 Department of Biosciences, Sri Sathya Sai Institute of Higher Learning, Prasanthi Nilayam, 515134 India; 4 Department of Zoology, Stockholm University, Svante Arrhenius väg 18B, S-106 91 Stockholm, Sweden

**Keywords:** Nutrition, Developmental plasticity, Environmental matching, Fecundity, Fat, Glycogen

## Abstract

**Background:**

The adaptive significance of phenotypic changes elicited by environmental conditions experienced early in life has long attracted attention in evolutionary biology. In this study, we used *Drosophila melanogaster* to test whether the developmental diet produces phenotypes better adapted to cope with similar nutritional conditions later in life. To discriminate among competing hypotheses on the underlying nature of developmental plasticity, we employed a full factorial design with several developmental and adult diets. Specifically, we examined the effects of early- and late-life diets (by varying their yeast and sugar contents) on reproductive fitness and on the amount of energy reserves (fat and glycogen) in two wild-caught populations.

**Results:**

We found that individuals that had developed on either low-yeast or high-sugar diet showed decreased reproductive performance regardless of their adult nutritional environment. The lower reproductive fitness might be caused by smaller body size and reduced ovariole number. Overall, these results are consistent with the silver spoon concept, which posits that development in a suboptimal environment negatively affects fitness-associated traits. On the other hand, the higher amount of energy reserves (fat) in individuals that had developed in a suboptimal environment might represent either an adaptive response or a side-effect of compensatory feeding.

**Conclusion:**

Our findings suggest that the observed differences in the adult physiology induced by early-life diet likely result from inevitable and general effects of nutrition on the development of reproductive and metabolic organs, rather than from adaptive mechanisms.

## Background

The environmental conditions experienced during development profoundly affect health and physiology in adult life. One of the critical factors determining adult fitness is developmental nutrition. The link between the prenatal diet and metabolic and heart diseases in later life has been demonstrated in diverse mammalian models. For example, restriction of nutrients during early development leads to an increased risk of obesity, type-2 diabetes, and coronary heart diseases [1–3]. This effect is explained by the so-called thrifty phenotype hypothesis [4, 5], which states that poor nutritional conditions during early development programs metabolism to cope with nutritional scarcity later in life. However, if the conditions improve during adulthood and food becomes more abundant, the adaptation to undernourishment becomes maladaptive, causing obesity, type-2 diabetes, and related metabolic disorders. Not only undernutrition but also maternal/neonatal obesogenic (i.e. promoting excessive fat accumulation) environment results in long-term changes in energy metabolism and increases the incidence of metabolic diseases at a later age [2, 6]. According to a more general concept of the predictive adaptive response (PAR) proposed by Gluckman and Hanson [7], adverse effects arise from a mismatch between early- and late-life environments. The circumstances experienced during development may serve as clues for future environmental conditions, and trigger an adaptive response. Consequently, a match between developmental and adult environments increases individual fitness, whereas a mismatch can have negative impacts on survival and reproduction [8]. Predictive adaptive responses are instances of adaptive developmental plasticity [9]. According to theoretical studies [10, 11], the evolution of PARs might be expected only in organisms that live in very predictable environments.

Both the thrifty phenotype and PAR hypothesis can be categorised as an environmental matching model [12], which posits that in a given environment, individuals that developed in this environment have higher fitness than those that developed under different conditions; fitness is maximised when there is a match between developmental and adult environment [12]. An alternative explanation to the environmental matching model is the so-called silver spoon effect - favourable developmental conditions improve traits that are positively associated with adult fitness [13]. As a result, individuals that developed in a poor nutritional environment always have lower fitness than individuals reared under optimal conditions; both groups have higher fitness in resource-rich environments (reviewed in [12]). Monaghan [12] completed PAR and silver spoon concepts by implementing two new conceptual variations (models). In the first model, fitness is always higher in good conditions; however, individuals grown in poor conditions have better performance in a suboptimal environment than individuals that had developed under optimal conditions, i.e. developmental plasticity has adaptive character in this case. However, unlike the environmental matching model, fitness is not maximised by a match between developmental and adult environment. According to the second model, individuals that had developed under poor conditions always have lower fitness, which declines with increasing quality of an environment [12].

Over the past several decades, the fruit fly *Drosophila melanogaster* has become an excellent model organism for studying homeostatic regulation and metabolic disorders (e.g. [14]). Like in humans, obesity and diseases such as diabetes can be induced in flies by both dietary and genetic dysregulations (reviewed in [15]). Numerous studies in *Drosophila* have shown that early developmental diet has significant long-term consequences for adult physiology and various fitness-related traits such as lifespan, reproduction or stress tolerance. For example, flies reared on a low yeast diet have reduced body size (e.g. [16–18]), lower fecundity [17], but higher cold resistance [19]. In addition, a yeast-poor larval diet extends lifespan and increases the amount of fat reserves in adults [20, 21]. Altogether, the composition of the developmental diet has far-reaching consequences for *Drosophila* fitness. Nevertheless, there are only a few experimental studies in fruit flies [22, 23], which had tested the adaptive significance of phenotypic changes caused by the developmental diet in the context of adult nutritional environments. It is therefore still an open question, whether such changes represent adaptive responses to the given developmental diet, as suggested, for example, by the PAR hypothesis, or rather are inevitable consequences of development under sub-optimal conditions.

In this study, we used two wild-caught outbred populations of *D. melanogaster* to test to what extent does a match, or a mismatch between developmental and adult nutritional environment affect individual fitness. In order to distinguish among various hypotheses on the character of plasticity induced by early-life nutrition, we employed a full factorial design with all combinations of three developmental and adult diets that differed in the macronutrient composition [12]. We separately tested the impact of yeast (the principal source of protein and sterols for fruit flies [24–26]) and sugar concentration in *Drosophila* diet on two fitness-related traits. In *Drosophila*, diets with either reduced yeast or increased sugar content prolong development, reduce egg-to-adult survival, and decrease female fecundity e.g. [24, 27, 28]; these imbalanced diets can be therefore considered suboptimal. The first trait we examined is the early reproductive performance (fecundity and egg-to-adult viability). In a natural environment, *Drosophila* adults have a very short life expectancy (less than a week) [29]. Therefore, early reproductive performance represents a reliable proxy for reproductive fitness [29, 30]. The second fitness-related trait we measured, the amount of energy reserves (fat and glycogen), plays an essential role in surviving periods of food shortage [31].

## Results

### Effects of developmental diet on early reproductive fitness

**Fig. 1:**
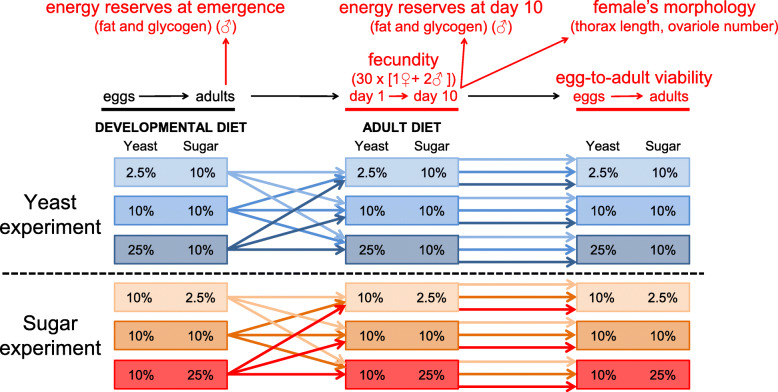
Full factorial experimental design with developmental and adult diets varying either in the yeast or sugar contents

To avoid the adverse effect of inbreeding on life-history traits (e.g. [32, 33]) and to account for potential population differences, we examined individuals from two outbred, wild-caught populations of *D. melanogaster* originating from temperate (Slovakia) and tropical climatic region (India). First, we analysed the effect of developmental and adult diets and their interactions on early fecundity (Fig. 1). Since both dietary yeast and sugar have a significant influence on fitness-associated traits (e.g. [24]), we analysed the effects of yeast and sucrose separately, using low (2.5%), intermediate (10%) and high (25%) concentrations of each compound.

Statistical analysis (multi-way ANOVA) of the effects of population origin (‘population’) and yeast concentration in the developmental and adult diet revealed that all these three factors, as well as their interactions (except for ‘population x developmental diet’), have significant impact on early fecundity (Additional file 1: Table S1). Similarly, the analysis of the effects of population and sugar content in the developmental and adult diet on early fecundity showed that all three factors and two of their interactions (‘population x adult diet’ and ‘population x developmental diet’) have a significant effect (Additional file 1: Table S2). In general, early fecundity was affected mainly by the adult diet; in comparison, the developmental diet had reduced importance, whereas population had only a minor effect. The dietary yeast and sugar had the opposite effect on reproductive output – while the early fecundity increased with the yeast content of the food, increased sugar concentration in the adult diet reduced the number of laid eggs.

**Fig. 2:**
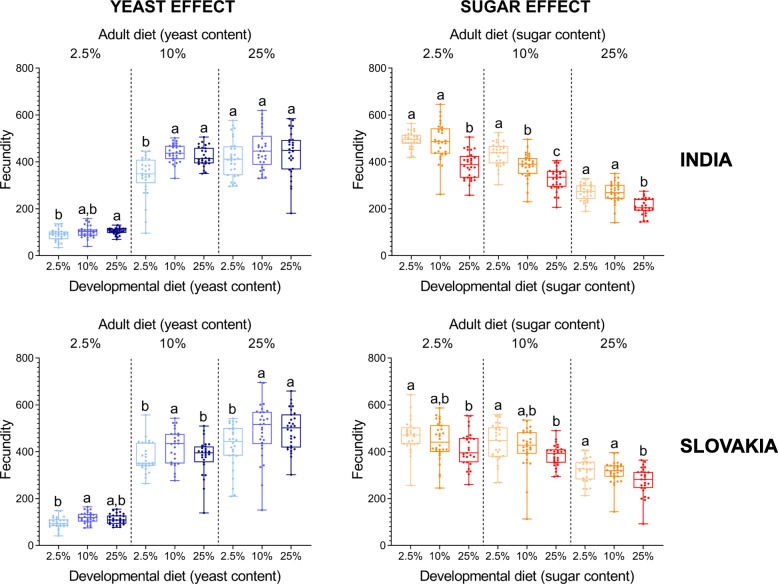
The developmental diet has a significant effect on early fecundity (sum of all eggs laid during the first ten days after emergence). Note that individuals from both Indian and Slovak populations that developed on the low-yeast diet tend to have lower fecundity irrespective of the adult diet. On the other hand, the high-sugar larval diet significantly decreases early fecundity in both populations regardless of the adult diet. Data for each population and adult diet were analysed by one-way ANOVA followed by Tukey’s HSD test (α = 0.05). Plots marked with different letters are significantly different from each other. Box plots depict minimum, first quartile, median, third quartile, and maximum value. For detailed statistical analyses, see Additional file 1: Tables S1 – S6

Although there were some minor population differences, individuals from both populations that had developed on the low-yeast diet (2.5% yeast) tended to lay (on average) fewer eggs on all three adult diets than individuals that had developed on either intermediate- or high-yeast diet (Fig. 2). Similarly, the flies that had developed on the high-sugar diet had the lowest early fecundity regardless of the sugar concentration in the adult diet (Fig. 2). Interestingly, a match between developmental and adult diets tended to have a relatively positive effect on early fecundity in the case of diets with varied yeast contents; however, a similar effect was not detected for dietary sugar (Additional file 1: Tables S3 and S4).

**Fig. 3:**
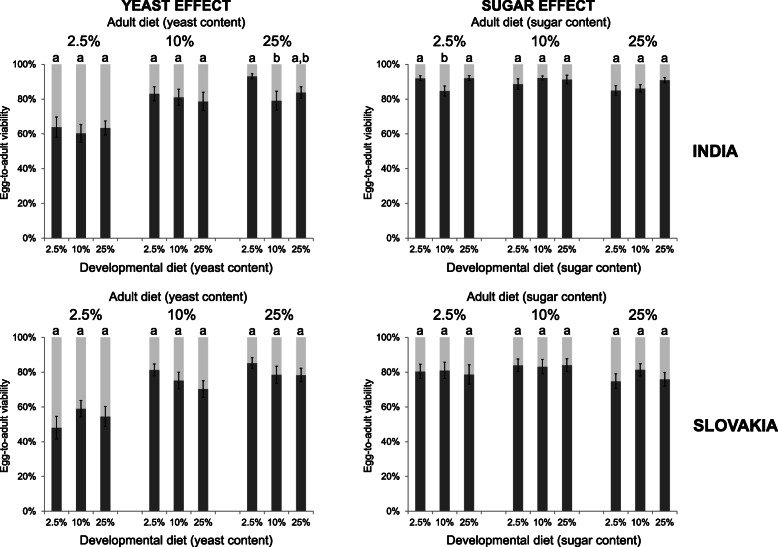
The developmental diet of parents has only a small or no effect on the egg-to-adult viability of offspring. Data for each population and adult diet were analysed by one-way ANOVA followed by Tukey’s HSD test (α = 0.05). Plots marked with different letters are significantly different from each other. Box plots depict minimum, first quartile, median, third quartile, and maximum value. For detailed statistical analyses, see Additional file 1: Tables S1-S6

Egg-to-adult viability was mainly unaffected by the developmental diet of parents; this trait was predominantly influenced by the medium on which the eggs were laid (Fig. 3; Additional file 1: Tables S1-S4).

Overall, we found that individuals that develop on either a low yeast or high sugar diet have reduced reproductive fitness independently of adult nutrition.

### Developmental diet and early fecundity standardised for body size and ovariole number

Early fecundity strongly positively correlates with body size and the number of ovarioles, the functional units producing eggs (e.g. [30, 34–36]). Since the developmental diet can affect both these traits, we investigated, whether the observed effects of the developmental diet on reproductive performance depend on changes in body size and ovariole number.

**Fig. 4:**
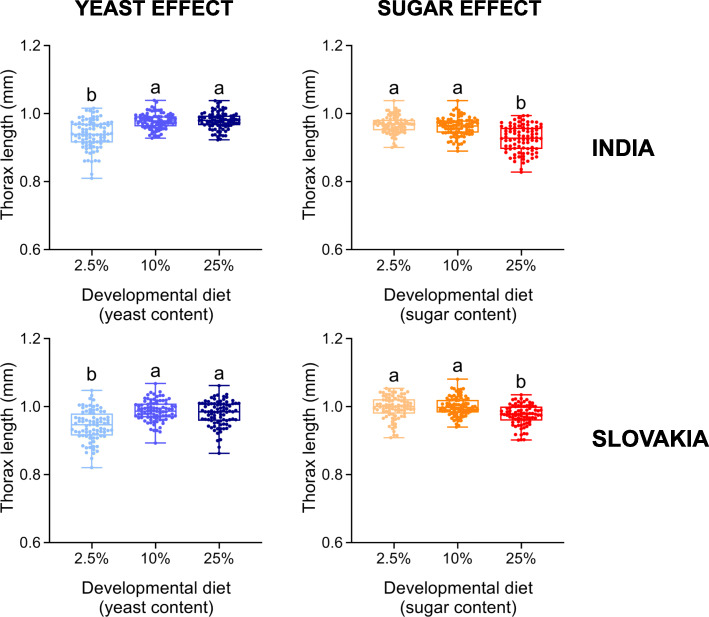
The yeast and sugar content in the developmental diet has a significant effect on thorax length. Individuals raised on the developmental diet with low (2.5%) yeast or with high (25%) sugar contents have reduced thorax length. Data for each population were analysed by one-way ANOVA followed by Tukey’s HSD test (α = 0.05). Plots marked with different letters are significantly different from each other. Box plots depict minimum, first quartile, median, third quartile, and maximum value. For detailed statistical analyses, see Additional file 1: Table S7

**Fig. 5:**
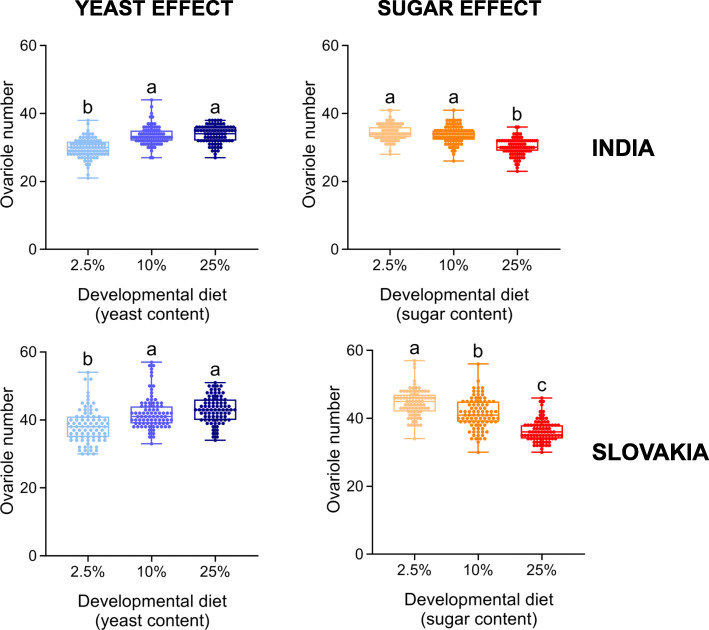
The yeast and sugar content in the developmental diet has a significant effect on ovariole number. Individuals raised on the developmental diet with low yeast (2.5%) or with high sugar (25%) contents have fewer ovarioles. Data for each population were analysed by one-way ANOVA followed by Tukey’s HSD test (α = 0.05). Plots marked with different letters are significantly different from each other. Box plots depict minimum, first quartile, median, third quartile, and maximum value. For detailed statistical analyses, see Additional file 1: Table S7

As expected, the yeast and sugar content of the developmental diet had pronounced effects on both thorax length and ovariole number (Figs. 4 and 5; Additional file 1: Table S7). Flies from both populations that had developed on the intermediate- and high-yeast diet were significantly larger and had more ovarioles than the individuals that had developed on the low-yeast diet (Figs. 4 and 5). The sugar content of the developmental diet had exactly inverse effects; individuals that had developed on the high-sugar diet were smaller and with significantly fewer ovarioles (Figs. 4 and 5).

**Fig. 6:**
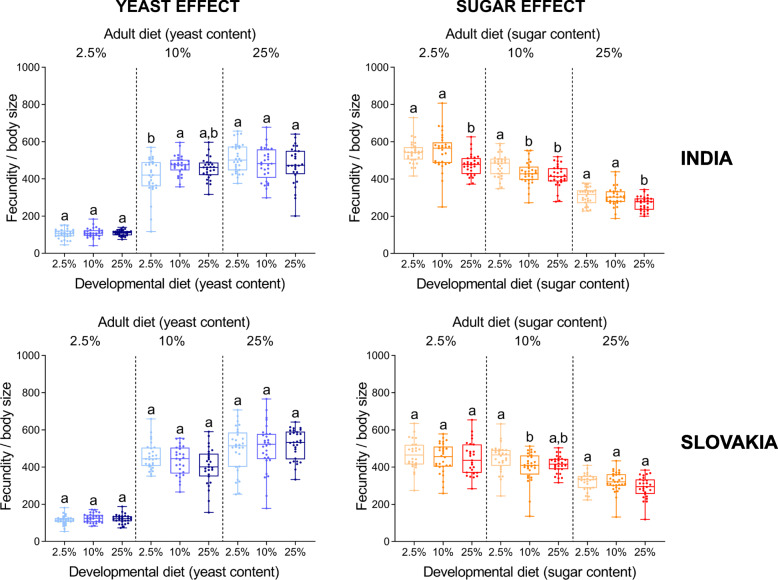
The developmental diet has only a small or no effect on early fecundity standardised for body size (thorax length^3^). Data for each group and population were analysed by one-way ANOVA followed by Tukey’s HSD test (α = 0.05). Plots marked with different letters are significantly different from each other. Box plots depict minimum, first quartile, median, third quartile, and maximum value. For detailed statistical analyses, see Additional file 1: Tables S1-S6

**Fig. 7:**
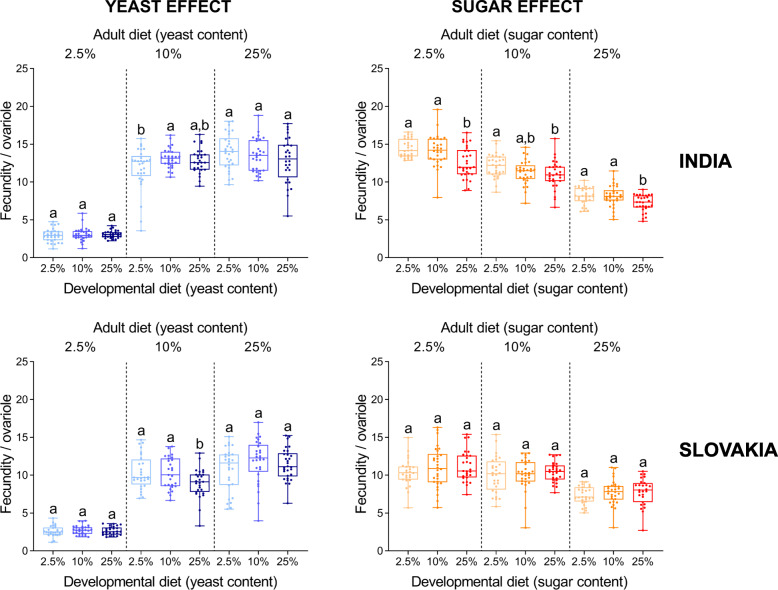
The yeast and sugar content in the developmental diet has a small or no effect on early fecundity per ovariole. Data for each group and population were analysed by one-way ANOVA followed by Tukey’s HSD test (α = 0.05). Plots marked with different letters are significantly different from each other. Box plots depict minimum, first quartile, median, third quartile, and maximum value. For detailed statistical analyses, see Additional file 1: Tables S1-S6

Next, we analysed to what extent the observed differences in early fecundity among the flies that had developed on different diets, are associated with variation in thorax length and ovariole number. We used two approaches: i) we standardised individual fecundity either by the body size (thorax length^3^) or ovariole number, and ii) we analysed fecundity by ANCOVA with either the thorax length or ovariole number as a covariate [37]. Importantly, both normalization methods provided similar results (Figs. 6 and 7; Additional file 1: Tables S8 and S9). When standardised for either body size or ovariole number, we detected only minor differences in the fecundity among flies that had developed on diets with varying amounts of yeast (Figs. 6 and 7). This might suggest that the significant differences in the reproductive performance can be explained by the diet-induced differences in body size and ovariole number. By contrast, although there were only minor differences in the fecundity standardised for either body size or ovariole number among the flies from Slovakia that had developed on diets with varied sugar contents, the negative impact of development on the high sugar diet on reproductive output was still observable in the population from India (Figs. 6 and 7). Overall, the fecundity standardised for either body size or ovariole number was affected primarily by adult diet. A match between developmental and adult diet did not seem to have a significant influence on these traits (Additional file 1: Tables S3 and S4).

In conclusion, we found that development on either low-yeast or high-sugar diet reduces body size and ovariole number, which might explain the observed lower reproductive fitness of individuals that developed on these diets.

### Effects of developmental diet on energy reserves

**Fig. 8:**
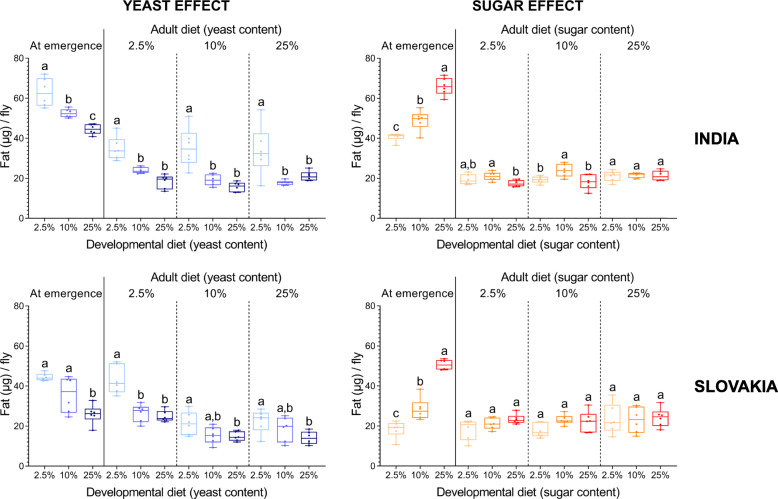
Effects of developmental diet on the amount of body fat in newly emerged and 10-day old individuals. Note that the flies that developed on the low-yeast diet have a significantly higher amount of fat stores irrespective of the adult diet; the sugar content in the developmental diet has only a small or no effect on the amount of body fat in 10-day old individuals. Data for each population and adult diet were analysed by one-way ANOVA followed by Tukey’s HSD test (α = 0.05). Plots marked with different letters are significantly different from each other. Box plots depict minimum, first quartile, median, third quartile, and maximum value. For a detailed statistical analysis, see Additional file 1: Tables S10-S12

**Fig. 9:**
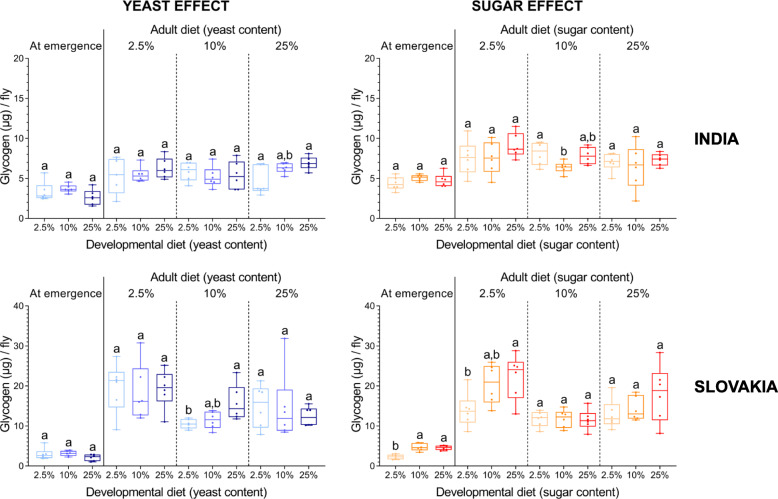
The yeast and sugar content in the developmental diet has only a small or no effect on the amount of glycogen either at emergence or in 10-day old individuals. Data for each population and adult diet were analysed by one-way ANOVA followed by Tukey’s HSD test (α = 0.05). Plots marked with different letters are significantly different from each other. Box plots depict minimum, first quartile, median, third quartile, and maximum value. For a detailed statistical analysis, see Additional file 1: Tables S10-S12

Finally, we examined how a match/mismatch between the developmental and adult diets affects the quantity of fat and glycogen reserves. We found that fat reserves are more sensitive to early life nutritional conditions than glycogen stores (Figs. 8 and 9; Additional file 1: Table S10). Development on the high-sugar or low-yeast diet significantly increased the fat content at emergence (Fig. 8).

The global statistical analysis showed that the three factors (‘population’, ‘developmental diet’ and ‘adult diet’), as well as some of their interactions, each have in most cases a significant effect on the fat and glycogen content ten days after emergence (Additional file 1: Tables S11 and S12). However, these effects were more pronounced in the case of fat reserves (Fig. 8) than glycogen reserves (Fig. 9). On diets with varying amounts of yeast, the developmental diet was the most important source of variation in the amount of fat reserves, whereas in the case of diets with varying amounts of sugar, the adult and developmental diet had comparable effects. Altogether, the flies that had developed on the yeast-poor diet had significantly higher fat content irrespective of adult nutrition (Fig. 8). Interestingly, the initially fatter flies that had developed on the high sugar diet had similar amounts of fat stores after ten days as flies that had developed on either intermediate- or low-sugar diet (Fig. 8). This suggests that unlike the low-yeast diet, the effect of the high-sugar developmental diet on body fat content does not persist. For the glycogen contents, we detected only small differences among flies that had developed on different diets (Fig. 9). Most of the variation in the amount of glycogen reserves was attributable primarily to the population origin and then to the adult diet. Importantly, we did not detect any significant effect of a match/mismatch between developmental and adult diets on either fat or glycogen reserves (Additional file 1: Tables S13 and S14).

In general, the adult diet had a similar effect on energy reserves as the developmental diet – the low-yeast and high-sugar diet increased the amount of fat stores. However, the differences in the body fat content among flies fed on the adult diets varying in the sugar content were rather small (Fig. 8). Since this observation was somewhat surprising, we conducted a follow-up experiment, in which we examined how these diets affect body fat content in two standard laboratory strains (Oregon R and *w*^*1118*^). This additional experiment revealed that variation in the sugar content of adult diet seems to have a relatively stronger effect on the amount of fat stores in the two laboratory strains than in the outbred wild-caught populations (Additional file 1: Fig. S1). This finding only confirms the previous results on the substantial intraspecific variation in response of lipid reserves to adult dietary sugar [38], and it may also point to a potential effect of inbreeding.

## Discussion

In this study, we tested different competing concepts, which try to explain how the interaction between early- and late-life nutritional environments affects individual fitness (reviewed in [12]). Based on our experimental data, individuals that had developed either on low-yeast or high-sugar diet had the lowest early reproductive performance. Despite developing in markedly different nutritional environments, both groups of flies shared many phenotypical similarities, such as prolonged development (not shown), smaller body size, fewer ovarioles and increased amount of fat reserves at emergence. Whether all these phenotypes are caused by a single factor (e.g. absolute (low-yeast diet) or relative (high-sugar diet) deficiency of proteins in the developmental diet) or are a result of suboptimal juvenile nutrition in general, may merit closer investigation.

According to the PAR hypothesis, fitness is maximised when the developmental and adult conditions match [7]. Although our results show that a match between developmental and adult diet might have a relatively positive effect on reproductive fitness, individuals that had developed on either low yeast or high sugar diet have lower reproductive fitness across all tested adult nutritional environments. Importantly, their early reproductive performance improved on more favourable adult diets. Overall, we did not find any evidence that individuals that had developed on low yeast or high sugar diet were better adapted to cope with such environments as adults. This finding is consistent with the silver spoon effect, which posits that individuals that developed in optimal conditions always have higher fitness than individuals that developed in a poor environment [13]. The silver spoon effect was also confirmed by other experimental studies that have tested the impact of a match/mismatch between developmental and adult environments on reproductive fitness. For example, Duxbury and Chapman [39] showed recently that fecundity of *Drosophila* is lower in females that have developed on a low-yeast diet. Similarly, May and Zwaan [23] also did not find evidence for the PAR hypothesis when examined *Drosophila* fecundity and lifespan. In this study, a low-calorie larval diet (low yeast and sugar content) increased both virgin lifespan and early fecundity (measured during the first seven days) irrespective of the adult diet; larval and adult diets had opposite effects on early fecundity. This discrepancy with our results (larval and adult diet have analogous effects on early fecundity) could be explained by the fact that in the study by May and Zwaan [23], yeast and sugar contents were manipulated together, whereas we have tested effects of these two components separately. If larvae are less tolerant to the high sugar concentration (due to glucose toxicity [40]) than adults, negative effects of increased sugar concentration in the larval diet might outweigh positive effects of increased yeast content. However, whether this is indeed the case requires further study.

Studies in other species also provide evidence for the silver spoon effect. For example, Barrett et al. [41] found in the cockroach *Nauphoeta cinerea* that poor juvenile diet reduced reproductive capacity irrespective of adult conditions. A similar effect has also been documented in the black field cricket *Teleogryllus commodus* [42], the ladybird beetle *Harmonia axyridis* [43] and the red flour beetle *Tribolium castaneum* [44]. In the butterfly *Bicyclus anynana*, females that experienced food limitation as larvae had lower early reproductive performance in both suboptimal and optimal adult nutritional environment, but only if they were not forced to fly. If they flew for 5 min, their fecundity was comparable to females that developed on an optimal diet [45]. The silver spoon effect might, therefore, be context-dependent (cf. [46]). In contrast, Taborsky [47] found that even though reproductive rate and offspring size in the cichlid fish *Simochromis pleurospilus* was influenced by juvenile diet, the reproductive success of females depended only on adult diet. Overall, it seems that the majority of experimental studies provide evidence that the developmental diet has indeed the silver spoon effect on reproductive fitness. However, one cannot exclude the possibility that the diet-induced changes might provide other fitness benefits, which cannot be easily assessed in a laboratory environment, e.g. lower energy investment in reproduction might allow for improved foraging abilities, and thus is adaptive for escaping from poor environments and finding more favourable conditions. Moreover, developmental diet affects also other traits, such as heat, cold, and desiccation resistance, and longevity (e.g. [19, 21, 23, 48]), which we did not examine in our study. It has been shown, for example, that poor-yeast larval diet increases cold resistance [19] and prolongs lifespan [20, 21, 23] – in natural environment, these traits might be perhaps more important in terms of individual fitness than early fecundity.

Another reason for not detecting adaptive plasticity might be that the developmental diets that reduced reproductive fitness represent stressful environments [49]. In other words, these nutritional conditions are unfavourable and not in the tolerance zone, but in the so-called ‘mitigation zone’, in which: “fitness declines as the development of an optimal phenotype is constrained” [12]. However, we presume that decreased reproductive performance on a low-yeast or high-sugar diet might be caused by reduced body size and ovariole number, traits that are positively associated with fecundity [30, 36, 50]. Since these morphological traits change gradually in response to yeast availability (Figs. 4 and 5), it is reasonable to assume that there is an optimal (mainly protein-dependent) developmental diet that maximises these two traits, and as a consequence also reproductive output. Of note, the existence of optimal early-life conditions has also been found for another environmental factor – developmental temperature (e.g. [37, 51, 52]).

The necessary condition for the evolution of predictive adaptive responses is that early-life environment carries reliable information about the future adult environment [53]. According to Barrett et al. [41], such condition might be fulfilled in insect species in which juveniles and adults share the same nutritional niche. In contrast, in species in which juveniles and adults differ significantly with regard to their diet, developmental conditions can hardly be indicative of adult conditions. Although *Drosophila* larvae and adults share a common nutritional niche (decaying fruit), it is questionable in this case to what extent can early-life environment provide a reliable clue for future conditions. Moreover, since adult *Drosophila* has relatively high mobility (its dispersal capacity is approximately 100–200 m per day [54], a distance that might be sufficient for finding suitable food resources), larval conditions are probably not a relevant indicator of the adult environment in fruit flies.

In holometabolous insects, a large proportion of energy reserves in young adults is accumulated during larval development [55]. Therefore, it is perhaps not surprising that the developmental diet has a significant impact on the amount of *Drosophila* energy stores at emergence. Specifically, a yeast-poor or sugar-rich larval diet increases the quantity of fat reserves in adults [20, 21, 56]. The inverse relation we observed here between the yeast content in the developmental diet and the amount of fat stored in 10-day old flies seems to support the thrifty phenotype hypothesis, i.e. poor nutritional conditions during early development can lead to increased accumulation of fat later in life [5]. Importantly, the higher fat content in adults that had developed on the low-yeast diet was not caused by increased fat deposition during adulthood (the overall amount of body fat declines with age), but by their larger fat reserves accumulated during the larval stage. As expected, flies that had developed on the high-sugar diet also stored substantially more fat at emergence (this amount was equal or higher than the amount of fat stored by flies that had developed on the low-yeast diet). However, after ten days, their fat content was comparable to flies that had developed on diets containing less sugar. Altogether, the ‘obese phenotype’ caused by a low-yeast larval diet seems to be more persistent than the one caused by a high-sugar larval diet. However, the underlying cause of this phenomenon is unclear. The increased amount of energy reserves in individuals that experienced poor larval conditions has also been documented in other insect species, such as the moth *Spodoptera eridania* [57], *Manduca sexta* [58], or the cockroach *Nauphoeta cinerea* [41]. The higher fat content in *Drosophila* individuals reared on a low-yeast diet leads to improved starvation resistance [21] – a quality which can be essential for survival in a nutrition-poor environment. Besides, a higher amount of energy reserves can increase dispersal capacity and allow moving to more favourable conditions [43]. Overall, the increased allocation of limited resources to energy reserves on a poor larval diet might represent a predictive adaptive response to anticipated adverse nutritional conditions in adulthood. On the other hand, undernutrition can potentially induce a fat phenotype also via compensatory feeding. Increased food intake as compensation for the deficiency of a crucial nutrient is a common physiological strategy (reviewed in [59]). In many species, including *Drosophila* [24] and humans [60], a low-protein diet increases food intake, which in turn leads to elevated energy uptake and promotes fat storage [24, 61]. Thus, although increased energy reserves provide undoubtedly an advantage in a poor nutritional environment, we presume that it is not an adaptive response, but more likely a consequence of compensatory feeding [21].

## Conclusions

The issue whether early developmental diet triggers adaptive phenotypic response is closely related to a more general and debatable question on the character of developmental plasticity (e.g. [62]). Whereas some traits may respond adaptively to environmental factors and stimuli, changes in others might be just inevitable consequences of the altered developmental environment [63]. Our data on the reproductive performance of *Drosophila* do not provide evidence for an adaptive character of diet-induced phenotypic changes; a match between developmental and adult nutritional conditions did not necessarily maximize reproductive fitness. Regardless of adult nutrition, development on either low-yeast or high-sugar diet negatively affected individual fecundity. The decreased performance resulted very likely from smaller body size and reduced ovariole number. These findings agree well with the notion of the silver spoon effect, i.e. unfavourable developmental conditions negatively affect fitness-related traits. On the other hand, increased accumulation of lipids during the larval stage in flies that developed on a low-yeast diet can certainly provide a substantial advantage in nutrient-poor conditions. However, whether this represents a predictive adaptive response or it is just a by-product of compensatory feeding as larvae merits further investigation.

## Methods

### Fly populations

We used two outbred wild-caught populations of *D. melanogaster* from (i) Slovakia (Bratislava; collected in October 2017), and (ii) India (Mysore, Karnataka; collected in July (monsoon season) 2017) (for further details see [37]). Both populations were kept in the laboratory as outbred (Indian population for approx. 28 and Slovakian population for approx. 24 generations prior to the experiments) at population sizes of approx. 1500–2000 adults with overlapping generations (generation time approx. 3 weeks). In addition, in a follow-up experiment, we also used two laboratory strains Oregon R and *w*^*1118*^. Prior to the experiments (see below), all flies were maintained on a standard *Drosophila* medium (6 g agar, 50 g yeast (instant dry yeast Backaldrin), 50 g sucrose, 70 g maize flour, 5.12 ml propionic acid and 1.3 g methylparaben per 1 l of medium) at 25 °C (12 h:12 h light/dark cycle, 60% humidity).

### Dietary treatments

The effect of the developmental diet on reproductive performance and the amount of energy reserves was tested by using full factorial design with three ‘developmental’ and three ‘adult’ diets (Fig. 1). To examine the effects of major dietary components, yeast and sugar, we carried out two separate experiments. In the first experiment, we used diets with different yeast concentration: low (2.5%), intermediate (10%), and high-yeast (25% yeast (instant dry yeast Backaldrin)) diet (with 10% sucrose, 6 g agar, 5.12 ml propionic acid and 1.3 g methylparaben per 1 l of medium). In the second experiment, we examined the effect of low (2.5%), intermediate (10%), and high-sugar (25% sucrose) diet (with 10% yeast, 6 g agar, 5.12 ml propionic acid and 1.3 g methylparaben per 1 l of medium) (Fig. 1). The choice of diets is based on the study by Skorupa et al. [24]. All experimental diets are within a range that *Drosophila* adults can tolerate without substantial reduction in lifespan [24].

### Fecundity and viability

To obtain parental flies for experimental individuals for measuring early reproductive performance, flies from stock populations laid eggs into vials (68 ml) with a standard *Drosophila* medium (22 ml) for four hours; afterwards, the adult flies were removed, and eggs were used to generate parental populations. Next, we enabled one-week-old parental flies (approx. 100 individuals) to lay eggs into vials with the distinct experimental diets (low, intermediate or high yeast/sugar diet) for four hours. In order to avoid larval crowding, we used an intermediate egg density – approx. 150 eggs per 68 ml vial (containing 22 ml of food); any excessive eggs were removed. Vials with eggs (12 vials per developmental diet per population) were kept at 25 °C, 12 h:12 h light/dark cycle, and 60% humidity. All experimental flies were collected within 12 h after emergence (females were collected as virgins) and randomly placed on three different ‘adult’ diets (low, intermediate or high yeast/sugar diet); i.e. for each population and the developmental diet, we established three different ‘adult’ diet groups (Fig. 1). Each group consisted of 30 females, which were kept individually (in 46 ml vials with 9 ml of food) with two males that developed on the same diet as females (i.e. the female fitness cannot technically be considered independent of paternal effects). All flies were transferred every 24 h to vials with fresh medium. The number of eggs was counted under a stereomicroscope. Early fecundity was expressed as a cumulative number of eggs laid by a single female during the first ten days [37]. After egg counting, vials with eggs laid on the 10th day were kept to determine the egg-to-adult viability – i.e. the proportion of eclosed flies. After ten days, all females were collected and examined for their thorax length and ovariole number. Data on females that escaped or died during the experiments were excluded from all analyses. At the end of the experiments, males were collected to determine their fat and glycogen content. All experiments were performed at 25 °C, 12 h:12 h light/dark cycle, and 60% humidity.

### Morphological traits

Thorax length was measured and the number of ovarioles determined as described in detail in [37]. Both morphological traits were measured in 10-day old females (i.e. 27–30 individuals per treatment per population), which were used in the fecundity and egg-to-adult viability assays (see the previous section Fecundity and viability). As a proxy for body size, we used the cubed thorax length (thorax length^3^) [37, 64]. Ovariole number is expressed as the sum of ovarioles from both ovaries.

### Fat and glycogen quantification

Energy reserves were quantified in newly emerged and 10-day old males (5–6 replicates per treatment per population; 5 males per replicate), which were used in the fecundity and egg-to-adult viability assays (see the section Fecundity and viability). Energy reserves were measured only in male flies, because, in females, a considerable proportion of triglycerides (fat) [65] and glycogen [66, 67] is allocated to the growing oocytes. Fat and glycogen content were determined using standard protocols based on the colourimetric assays [68]. Both types of energy reserves were measured from the same starting homogenates, prepared as described in detail in [69]. Lipids were quantified using the Triglycerides kit (Randox, TR1697). Glycogen levels were analysed by glucose oxidation kit (Sigma, GAGO20) after digestion by α-amyloglucosidase (Sigma A1602), as described previously [68].

In a follow-up experiment, we measured fat content in males (including the two laboratory strains - Oregon-R and *w*^*1118*^) that had developed on the intermediate sugar diet (10% sucrose). After emergence, they were transferred together with females (30 males and 30 females per vial/ replicate; 3 replicates per adult diet) either to low (5%), intermediate (10%), or high (25% sucrose) sugar diet with daily food exchange. After ten days, all males were collected (5–6 replicates per treatment per population/strain; 5 males per replicate), and their lipid content quantified as described above.

### Statistical analysis

We performed a global analysis of individual fitness-related traits (early fecundity, viability, early fecundity standardised for body size, early fecundity per ovariole number, fat and glycogen content) by using full factorial, multi-way analysis of variance (ANOVA, type III sum of squares) with population, developmental and adult diet as fixed effects, and their interactions. To examine the effect of population and the developmental diet on a given trait at different adult diets, we used full factorial, two-way fixed-effects ANOVA with interaction. To test the effect of the developmental diet on a given trait at different adult nutrition and separately for each population, we performed one-way ANOVA with Tukey’s Honest Significant Difference (HSD) posthoc test with α = 0.05. Thorax length and ovariole number were analysed by using two-way ANOVA with two fixed factors, population and the developmental diet, and their interaction, and also separately for each population by one-way ANOVA with the developmental diet as a fixed factor with Tukey’s HSD posthoc test (α = 0.05). To control for differences in body size and ovariole number, we used the same approach as described in [37]. In addition to analysing fecundity standardised for body size (thorax length^3^) and fecundity per ovariole, we performed for each population and adult diet analysis of covariance (ANCOVA), with the developmental diet as the fixed factor and thorax length or ovariole number as the covariate, followed by Tukey’s HSD posthoc test (α = 0.05). Finally, to test whether a match vs mismatch between developmental and adult diet affects a given trait, first, we examined the residuals from the ANOVA (using all factors - population, developmental and adult diet) dependent on the degree of mismatch (similar = 0 [larval 2.5% (either yeast or sugar) and adult 2.5%, larval 10% and adult 10%, larval 25% and adult 25%], one step dissimilar = 1 [e.g. larval 2.5% and adult 10%, larval 10% and adult 25% etc.], two steps dissimilar = 2 [e.g. larval 2.5% and adult 25% and larval 25% and adult 2.5%]. If the residuals of the model were highest for 0, intermediate for 1 and lowest for 2, this would indicate that a match between nutritional environments has a positive effect on a given trait. Next, we fitted the degree of mismatch (0, 1, 2) in the ANOVA model with all factors (population, developmental diet, adult diet, and mismatch).

All analyses were performed using JMP 15 (SAS Institute, Cary, NC, USA) and PAST 3.11 software [70]; graphs were created in Graphpad Prism 8 (GraphPad Software Inc.) and Excel (Microsoft).

## Supplementary information

**Additional file 1: Fig. S1**. Intraspecific variation in the effect of sugar in the adult diet on the amount of fat in 10 day-old males. **Table S1.** The results of ANOVA for the effect of population, the yeast content in the developmental and the adult diet, and their interactions on early fecundity, egg-to-adult viability, early fecundity standardised for body size, and early fecundity per ovariole. **Table S2.** The results of ANOVA for the effect of population, the sugar content in the developmental and adult diet, and their interactions on early fecundity, egg-to-adult viability, early fecundity standardised for body size, and early fecundity per ovariole. **Table S3.** The results of ANOVA for the effect of population, the yeast content in the developmental and the adult diet, and a match/mismatch between developmental and adult nutritional environment on early fecundity, egg-to-adult viability, early fecundity standardised for body size, and early fecundity per ovariole. **Table S4.** The results of ANOVA for the effect of population, the sugar content in the developmental and the adult diet, and a match/mismatch between developmental and adult nutritional environment on early fecundity, egg-to-adult viability, early fecundity standardised for body size, and early fecundity per ovariole. **Table S5.** The results of ANOVA for the effect of population and the yeast content in the developmental diet, and their interactions on various traits on different adult diets. **Table S6.** The results of ANOVA for the effect of population and the sugar content in the developmental diet, and their interactions on various traits on different adult diets. **Table S7.** The results of ANOVA for the effect of population and the yeast or sugar content in the developmental diet, and their interactions on thorax length and ovariole number. **Table S8.** The ANCOVA results for early fecundity with the yeast content in the developmental diet as the fixed factor and thorax length or ovariole number as the covariate. **Table S9.** The ANCOVA results for early fecundity with the sugar content in the developmental diet as the fixed factor and thorax length or ovariole number as the covariate. **Table S10.** The results of ANOVA for the effect of population and the yeast or sugar content in the developmental diet, and their interactions on the fat and glycogen content at emergence. **Table S11.** The results of ANOVA for the effect of population, the yeast content in the developmental and the adult diet, and their interactions on the fat content and glycogen content in 10-day old males. **Table S12.** The results of ANOVA for the effect of population, the sugar content in the developmental and the adult diet, and their interactions on fat content and glycogen content in 10-day old males. **Table S13.** The results of ANOVA for the effect of population, the yeast content in the developmental and the adult diet, and a match/mismatch between developmental and adult nutritional environment on fat content and glycogen content in 10-day old males. **Table S14.** The results of ANOVA for the effect of population, the sugar content in the developmental and the adult diet, and a match/mismatch between developmental and adult nutritional environment on fat content and glycogen content in 10-day old males.

**Additional file 2.** Data. Data generated and analysed during this study.

## Data Availability

All data generated and analysed during this study are included in this article and its supplementary information files.

## Abbreviations

ANCOVA: Analysis of covariance
ANOVA: Analysis of variance
PAR: Predictive adaptive response
Tukey’s HSD test: Tukey’s honest significant difference test
