# Supplementary material for: The influence of developmental diet on reproduction and metabolism in Drosophila
Source: BMC Evol Biol. 2020 Jul 29;20:93. doi: 10.1186/s12862-020-01663-y (PMC7392729; doi:10.1186/s12862-020-01663-y)
Supplement: Supplementary file 1 — Additional file 1: Fig. S1. Intraspecific variation in the effect of sugar in the adult diet on the amount of fat in 10 day-old males. Table S1. The results of ANOVA for the effect of population, the yeast content in the developmental and the adult diet, and their interactions on early fecundity, egg-to-adult viability, early fecundity standardised for body size, and early fecundity per ovariole. Table S2. The results of ANOVA for the effect of population, the sugar content in the developmental and adult diet, and their interactions on early fecundity, egg-to-adult viability, early fecundity standardised for body size, and early fecundity per ovariole. Table S3. The results of ANOVA for the effect of population, the yeast content in the developmental and the adult diet, and a match/mismatch between developmental and adult nutritional environment on early fecundity, egg-to-adult viability, early fecundity standardised for body size, and early fecundity per ovariole. Table S4. The results of ANOVA for the effect of population, the sugar content in the developmental and the adult diet, and a match/mismatch between developmental and adult nutritional environment on early fecundity, egg-to-adult viability, early fecundity standardised for body size, and early fecundity per ovariole. Table S5. The results of ANOVA for the effect of population and the yeast content in the developmental diet, and their interactions on various traits on different adult diets. Table S6. The results of ANOVA for the effect of population and the sugar content in the developmental diet, and their interactions on various traits on different adult diets. Table S7. The results of ANOVA for the effect of population and the yeast or sugar content in the developmental diet, and their interactions on thorax length and ovariole number. Table S8. The ANCOVA results for early fecundity with the yeast content in the developmental diet as the fixed factor and thorax length or ovariole number as the covariat [file 12862_2020_1663_MOESM1_ESM.pdf]

Supplementary Information

The influence of developmental diet on reproduction and metabolism in *Drosophila*

Peter Klepsatel, Diana Knoblochová, Thirnahalli Nagaraj Girish, Heinrich Dircksen and Martina Gáliková

Supporting Figure Legends

Figure S1. Intraspecific variation in the effect of sugar in the adult diet on the amount of fat in 10 day-old males. Data for each population/strain were analysed by one-way ANOVA followed by Tukey's HSD test ($\alpha = 0.05$). Plots marked with different letters are significantly different from each other. Box plots depict minimum, first quartile, median, third quartile, and maximum value. For details, see Material and methods.

Figure S1

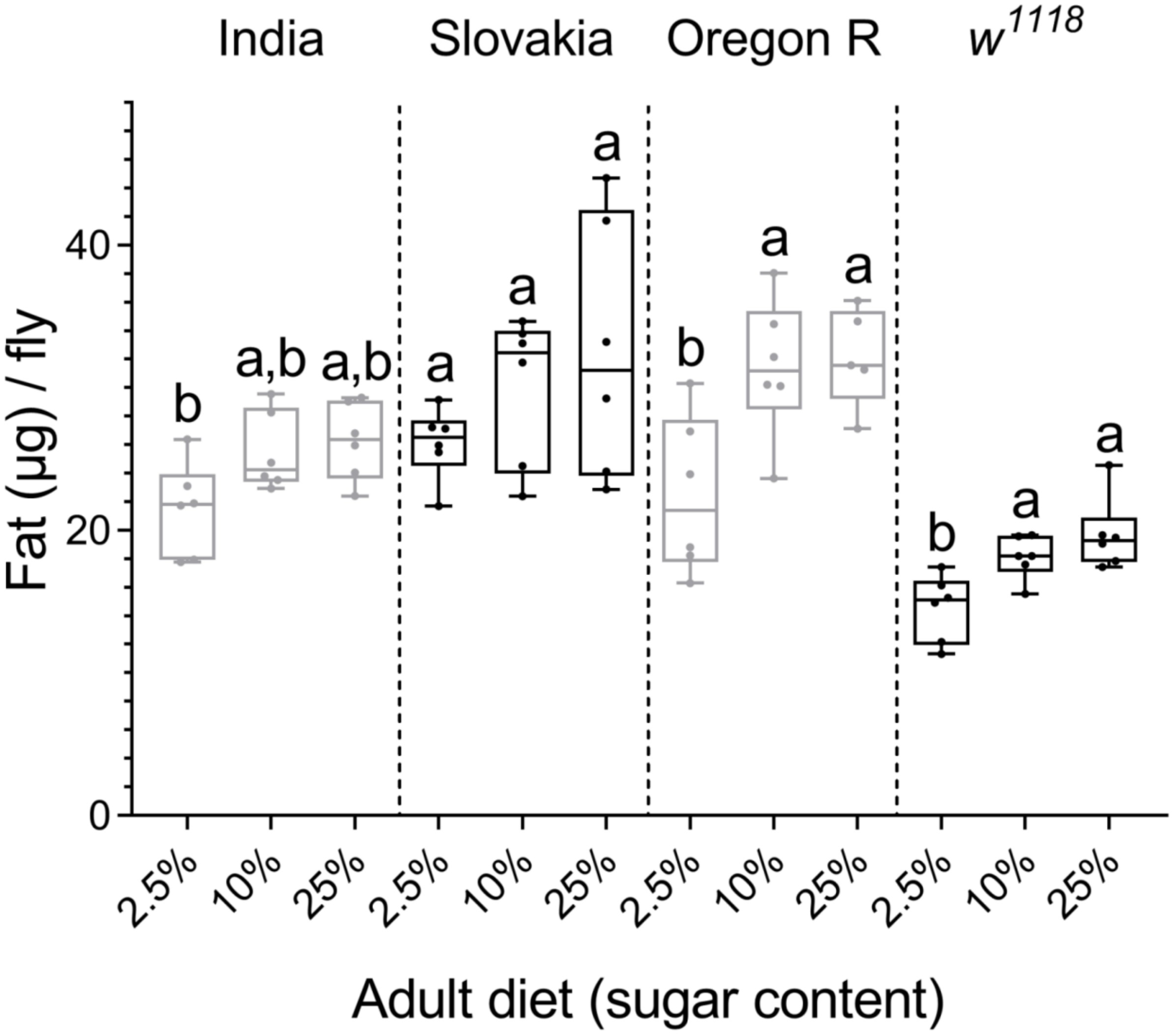

India
Slovakia
Oregon R
w^{1118}
Fat (µg) / fly
0
20
40
b
a,b
a,b
a
a
a
b
a
a
b
a
a
2.5%
10%
25%
2.5%
10%
25%
2.5%
10%
25%
2.5%
10%
25%
Adult diet (sugar content)

Supplementary Tables

Table S1. The results of ANOVA for the effect of population, the **yeast content** in the developmental and the adult diet, and their interactions on early fecundity, egg-to-adult viability, early fecundity standardised for body size, and early fecundity per ovariole. *df* - degrees of freedom; SSQ - the sum of squares for each source of variation.

Trait	Source of variation	*df*	SSQ	*F*-ratio	*P*-value
Early fecundity	Population	1	18902	4.10	0.043
	Developmental diet	2	199258	21.63	< 0.0001
	Adult diet	2	12106730	1314.13	< 0.0001
	Population × Developmental diet	2	1718	0.19	0.830
	Population × Adult diet	2	40276	4.37	0.013
	Developmental diet × Adult diet	4	45131	2.45	0.045
	Population × Developmental diet × Adult diet	4	60691	3.29	0.011
	Error	497	2289371	-	-
Egg-to-adult viability	Population	1	0.49	8.20	0.004
	Developmental diet	2	0.18	1.47	0.232
	Adult diet	2	5.96	49.61	< 0.0001
	Population × Developmental diet	2	0.09	0.72	0.490
	Population × Adult diet	2	0.04	0.33	0.716
	Developmental diet × Adult diet	4	0.37	1.53	0.193
	Population × Developmental diet × Adult diet	4	0.13	0.56	0.696
	Error	492	29.53	-	-
Early fecundity standardised for body size	Population	1	4190	0.72	0.396
	Developmental diet	2	4667	0.40	0.669
	Adult diet	2	14845177	1280.39	< 0.0001
	Population × Developmental diet	2	2945	0.25	0.776
	Population × Adult diet	2	28183	2.43	0.089
	Developmental diet × Adult diet	4	19629	0.85	0.496
	Population × Developmental diet × Adult diet	4	102027	4.40	0.002
	Error	497	2881184	-	-
Early fecundity per ovariole	Population	1	388.48	102.86	< 0.0001
	Developmental diet	2	21.21	2.81	0.061
	Adult diet	2	9328.50	1235.02	< 0.0001
	Population × Developmental diet	2	1.83	0.24	0.785
	Population × Adult diet	2	131.15	17.36	< 0.0001
	Developmental diet × Adult diet	4	9.26	0.61	0.653
	Population × Developmental diet × Adult diet	4	66.90	4.43	0.002
	Error	496	1873.22	-	-

Table S2. The results of ANOVA for the effect of population, the **sugar content** in the developmental and adult diet, and their interactions on early fecundity, egg-to-adult viability, early fecundity standardised for body size, and early fecundity per ovariole. *df* - degrees of freedom; SSQ - the sum of squares for each source of variation.

**Trait**	**Source of variation**	***df***	**SSQ**	***F*-ratio**	***P*-value**
Early fecundity	Population	1	72658.6	19.75	< 0.0001
	Developmental diet	2	483997.4	65.77	< 0.0001
	Adult diet	2	2651179.5	360.26	< 0.0001
	Population × Developmental diet	2	40361.2	5.49	0.004
	Population × Adult diet	2	105291.4	14.31	< 0.0001
	Developmental diet × Adult diet	4	23363.8	1.59	0.176
	Population × Developmental diet × Adult diet	4	20199.4	1.37	0.242
	Error	499	1836100.6	-	-
Egg-to-adult viability	Population	1	1.00	32.41	< 0.0001
	Developmental diet	2	0.012	0.24	0.785
	Adult diet	2	0.20	3.26	0.039
	Population × Developmental diet	2	0.08	1.27	0.282
	Population × Adult diet	2	0.02	0.37	0.694
	Developmental diet × Adult diet	4	0.081	0.64	0.636
	Population × Developmental diet × Adult diet	4	0.09	0.69	0.602
	Error	496	15.35	-	-
Early fecundity standardised for body size	Population	1	66837.3	15.18	0.0001
	Developmental diet	2	146189.5	16.61	< 0.0001
	Adult diet	2	3086759.8	350.61	< 0.0001
	Population × Developmental diet	2	28127.4	3.20	0.042
	Population × Adult diet	2	163486.6	18.57	< 0.0001
	Developmental diet × Adult diet	4	37745.9	2.14	0.074
	Population × Developmental diet × Adult diet	4	15170.5	0.86	0.487
	Error	499	2196583.7	-	-
Early fecundity per ovariole	Population	1	271.89	86.60	< 0.0001
	Developmental diet	2	16.86	2.69	0.069
	Adult diet	2	1871.85	298.11	< 0.0001
	Population × Developmental diet	2	78.27	12.47	< 0.0001
	Population × Adult diet	2	157.53	25.09	< 0.0001
	Developmental diet × Adult diet	4	12.32	0.98	0.417
	Population × Developmental diet × Adult diet	4	8.69	0.69	0.598
	Error	499	1566.60	-	-

Table S3. The results of ANOVA for the effect of population, the **yeast content** in the developmental and the adult diet, and a match/mismatch between developmental and adult nutritional environment on early fecundity, egg-to-adult viability, early fecundity standardised for body size, and early fecundity per ovariole. *df* - degrees of freedom; SSQ - the sum of squares for each source of variation.

Trait	Source of variation	*df*	SSQ	*F*-ratio	*P*-value
Early fecundity	Population	1	20391	4.31	0.039
	Developmental diet	2	186422	19.68	< 0.0001
	Adult diet	2	11854545	1251.57	< 0.0001
	Mismatch	2	36231	3.83	0.022*
	Error	507	2401095	-	-
Egg-to-adult viability	Population	1	0.47	7.89	0.005
	Developmental diet	2	0.164	1.31	0.272
	Adult diet	2	6.01	50.33	< 0.0001
	Mismatch	2	0.18	1.48	0.230
	Error	502	29.97	-	-
Early fecundity standardised for body size	Population	1	5129	0.86	0.354
	Developmental diet	2	12735	1.07	0.344
	Adult diet	2	14623209	1228.54	< 0.0001
	Mismatch	2	16470	1.38	0.252
	Error	507	3017385	-	-
Early fecundity per ovariole	Population	1	381.35	93.08	< 0.0001
	Developmental diet	2	28.58	3.49	0.031
	Adult diet	2	9208.13	1123.77	< 0.0001
	Mismatch	2	7.26	0.89	0.413
	Error	506	2073.07	-	-

* Comparison of residuals: 0 (11.83 ± 5.25 SEM) > 2 (-4.27±6.36) > 1 (-6.62 ±4.53)

Table S4. The results of ANOVA for the effect of population, the **sugar content** in the developmental and the adult diet, and a match/mismatch between developmental and adult nutritional environment on early fecundity, egg-to-adult viability, early fecundity standardised for body size, and early fecundity per ovariole. *df* - degrees of freedom; SSQ - the sum of squares for each source of variation.

**Trait**	**Source of variation**	***df***	**SSQ**	***F*-ratio**	***P*-value**
Early fecundity	Population	1	71777.2	18.22	< 0.0001
	Developmental diet	2	468880.8	59.51	< 0.0001
	Adult diet	2	2617852.8	332.25	< 0.0001
	Mismatch	2	21894.5	2.78	0.063*
	Error	509	2005219.6	-	-
Egg-to-adult viability	Population	1	0.99	32.24	< 0.0001
	Developmental diet	2	0.02	0.29	0.747
	Adult diet	2	0.15	2.35	0.096
	Mismatch	2	0.03	0.50	0.607
	Error	506	15.59	-	-
Early fecundity standardised for body size	Population	1	67226.1	14.2209	0.0002
	Developmental diet	2	151038.7	15.9752	< 0.0001
	Adult diet	2	3054743.2	323.0969	< 0.0001
	Mismatch	2	35966.8	3.8042	0.023**
	Error	509	2406188.4	-	-
Early fecundity per ovariole	Population	1	271.74	76.43	< 0.0001
	Developmental diet	2	16.22	2.28	0.1033
	Adult diet	2	1874.67	263.64	< 0.0001
	Mismatch	2	12.52	1.76	0.173
	Error	509	1809.66	-	-

*Comparison of residuals: 1 (3.95 ± 4.09) > 0 (1.09 ± 4.81) > 2 (-9.72 ± 5.86)
** Comparison of residuals: 1 (8.00 ± 4.48) > 0 (-6.44 ± 5.27) > 2 (-6.88 ± 6.42)

Table S5. The results of ANOVA for the effect of population and the **yeast content** in the developmental diet, and their interactions on various traits on different adult diets. *df* - degrees of freedom; SSQ - the sum of squares for each source of variation.

Trait	Adult diet	Source of variation	*df*	SSQ	*F*-ratio	*P*-value
Early fecundity	2.5% yeast	Population	1	4964.22	9.55	0.002
		Developmental diet	2	9895.71	9.51	0.0001
		Population × Developmental diet	2	630.67	0.61	0.547
		Error	168	87368.47	-	-
	10% yeast	Population	1	3270.16	0.750	0.388
		Developmental diet	2	140918.69	16.153	< 0.0001
		Population × Developmental diet	2	42866.97	4.914	0.009
		Error	161	702267.01	-	-
	25% yeast	Population	1	51857.10	5.81	0.017
		Developmental diet	2	92924.45	5.20	0.006
		Population × Developmental diet	2	18610.12	1.04	0.355
		Error	168	1499735.2	-	-
Egg-to-adult viability	2.5% yeast	Population	1	0.32	4.03	0.046
		Developmental diet	2	0.04	0.27	0.763
		Population × Developmental diet	2	0.15	0.93	0.396
		Error	166	13.36	-	-
	10% yeast	Population	1	0.12	2.09	0.150
		Developmental diet	2	0.16	1.46	0.235
		Population × Developmental diet	2	0.03	0.27	0.761
		Error	159	8.81	-	-
	25% yeast	Population	1	0.09	2.13	0.146
		Developmental diet	2	0.34	3.85	0.023
		Population × Developmental diet	2	0.04	0.45	0.638
		Error	167	7.36	-	-
Early fecundity standardised for body size	2.5% yeast	Population	1	7080.53	12.07	0.0007
		Developmental diet	2	2062.87	1.76	0.176
		Population × Developmental diet	2	262.74	0.22	0.800
		Error	168	98588.26	-	-
	10% yeast	Population	1	9403.06	1.45	0.231
		Developmental diet	2	18024.84	1.39	0.253
		Population × Developmental diet	2	73195.37	5.63	0.004
		Error	161	1046942.6	-	-
	25% yeast	Population	1	16314.93	1.58	0.211
		Developmental diet	2	3831.39	0.19	0.831
		Population × Developmental diet	2	31162.88	1.51	0.224
		Error	168	1735653.3	-	-
Early fecundity per ovariole	2.5% yeast	Population	1	5.54	11.31	0.001
		Developmental diet	2	0.49	0.50	0.610
		Population × Developmental diet	2	0.07	0.07	0.928
		Error	167	81.851	-	-
	10% yeast	Population	1	316.40	74.51	< 0.0001
		Developmental diet	2	20.67	2.43	0.091
		Population × Developmental diet	2	39.32	4.63	0.011
		Error	161	683.71	-	-
	25% yeast	Population	1	193.12	29.29	< 0.0001
		Developmental diet	2	8.72	0.66	0.518
		Population × Developmental diet	2	29.05	2.20	0.114
		Error	168	1107.67	-	-

Table S6. The results of ANOVA for the effect of population and the **sugar content** in the developmental diet, and their interactions on various traits on different adult diets. *df* - degrees of freedom; SSQ - the sum of squares for each source of variation.

Trait	Adult diet	Source of variation	*df*	SSQ	*F*-ratio	*P*-value
Early fecundity	2.5% sugar	Population	1	9254.83	1.93	0.167
		Developmental diet	2	223530.80	23.32	< 0.0001
		Population × Developmental diet	2	35671.06	3.72	0.026
		Error	166	795514.8	-	-
	10% sugar	Population	1	44295.13	10.79	0.001
		Developmental diet	2	184782.42	22.51	< 0.0001
		Population × Developmental diet	2	22130.78	2.70	0.071
		Error	166	681460.39	-	-
	25% sugar	Population	1	124862.92	58.06	< 0.0001
		Developmental diet	2	100485.85	23.36	< 0.0001
		Population × Developmental diet	2	3253.62	0.76	0.471
		Error	167	359125.40	-	-
Egg-to-adult viability	2.5% sugar	Population	1	0.39	10.39	0.002
		Developmental diet	2	0.03	0.46	0.630
		Population × Developmental diet	2	0.08	1.02	0.365
		Error	166	6.26	-	-
	10% sugar	Population	1	0.21	7.47	0.007
		Developmental diet	2	0.01	0.13	0.882
		Population × Developmental diet	2	0.011	0.25	0.783
		Error	164	4.54	-	-
	25% sugar	Population	1	0.43	15.61	0.0001
		Developmental diet	2	0.05	0.95	0.388
		Population × Developmental diet	2	0.08	1.39	0.253
		Error	166	4.56	-	-
Early fecundity standardised for body size	2.5% sugar	Population	1	199980.58	31.43	< 0.0001
		Developmental diet	2	61419.77	4.83	0.009
		Population × Developmental diet	2	36926.78	2.90	0.058
		Error	166	1056164.9	-	-
	10% sugar	Population	1	14842.07	3.41	0.067
		Developmental diet	2	80620.18	9.25	0.0002
		Population × Developmental diet	2	5857.54	0.67	0.512
		Error	166	723019.97	-	-
	25% sugar	Population	1	14930.74	5.97	0.016
		Developmental diet	2	42645.98	8.53	0.0003
		Population × Developmental diet	2	614.80	0.12	0.884
		Error	167	417398.91	-	-
Early fecundity per ovariole	2.5% sugar	Population	1	355.50	86.14	< 0.0001
		Developmental diet	2	17.07	2.07	0.130
		Population × Developmental diet	2	54.94	6.66	0.002
		Error	166	685.06	-	-
	10% sugar	Population	1	71.27	21.17	< 0.0001
		Developmental diet	2	6.67	0.99	0.374
		Population × Developmental diet	2	17.00	2.53	0.083
		Error	166	558.94	-	-
	25% sugar	Population	1	1.52	0.79	0.376
		Developmental diet	2	5.66	1.47	0.234
		Population × Developmental diet	2	14.91	3.86	0.023
		Error	167	322.60	-	-

Table S7. The results of ANOVA for the effect of population and the **yeast or sugar content** in the developmental diet, and their interactions on thorax length and ovariole number. *df* - degrees of freedom; SSQ - the sum of squares for each source of variation.

Dietary component	Trait	Source of variation	*df*	SSQ	*F*-ratio	*P*-value
Yeast	Thorax length	Population	1	0.003	2.89	0.090
		Developmental diet	2	0.18	74.96	< 0.0001
		Population × Developmental diet	2	0.001	0.57	0.568
		Error	509	0.61	-	-
	Ovariole number	Population	1	9973.47	696.19	< 0.0001
		Developmental diet	2	2017.89	70.43	< 0.0001
		Population × Developmental diet	2	13.03	0.45	0.635
		Error	508	7277.53	-	-
Sugar	Thorax length	Population	1	0.20	215.67	< 0.0001
		Developmental diet	2	0.11	59.02	< 0.0001
		Population × Developmental diet	2	0.01	4.76	0.009
		Error	511	0.48	-	-
	Ovariole number	Population	1	8201.63	750.69	< 0.0001
		Developmental diet	2	3575.84	163.65	< 0.0001
		Population × Developmental diet	2	322.52	14.76	< 0.0001
		Error	511	5582.88	-	-

Table S8. The ANCOVA results for early fecundity with the **yeast content** in the developmental diet as the fixed factor and thorax length or ovariole number as the covariate. *df* - degrees of freedom; SSQ - the sum of squares for each source of variation.

Adult diet	Population	Source of variation	*df*	SSQ	*F*-ratio	*P*-value	Source of variation	*df*	SSQ	*F*-ratio	*P*-value
2.5% yeast	India	Developmental diet	2	422.89	0.49	0.615	Developmental diet	2	422.89	0.49	0.615
		Thorax length	1	5318.31	12.31	0.0007	Ovariole number	1	5318.31	12.31	0.0007
		Error	85	36732.97	-	-	Error	85	36732.97	-	-
	Slovakia	Developmental diet	2	2513.43	2.60	0.081	Developmental diet	2	3166.69	2.89	0.061
		Thorax length	1	6142.72	12.70	0.0006	Ovariole number	1	815.12	1.49	0.226
		Error	81	39174.46	-	-	Error	80	43839.02	-	-
10% yeast	India	Developmental diet	2	83109.84	12.02	< 0.0001	Developmental diet	2	83109.84	12.02	< 0.0001
		Thorax length	1	13301.22	3.85	0.053	Ovariole number	1	13301.22	3.85	0.053
		Error	81	279994.23	-	-	Error	81	279994.23	-	-
	Slovakia	Developmental diet	2	20077.33	1.99	0.143	Developmental diet	2	29072.29	2.79	0.068
		Thorax length	1	15917.88	3.16	0.079	Ovariole number	1	1966.26	0.38	0.541
		Error	78	393053.68	-	-	Error	78	407005.30	-	-
25% yeast	India	Developmental diet	2	5798.61	0.43	0.654	Developmental diet	2	5798.61	0.43	0.654
		Thorax length	1	79798.87	11.77	0.0009	Ovariole number	1	79798.87	11.77	0.0009
		Error	83	562799.75	-	-	Error	83	562799.75	-	-
	Slovakia	Developmental diet	2	31619.85	1.59	0.209	Developmental diet	2	74309.01	3.63	0.031
		Thorax length	1	34000.24	3.43	0.068	Ovariole number	1	8139.57	0.80	0.375
		Error	83	823136.31	-	-	Error	83	848996.98	-	-

Table S9. The ANCOVA results for early fecundity with the **sugar content** in the developmental diet as the fixed factor and thorax length or ovariole number as the covariate. *df* - degrees of freedom; SSQ - the sum of squares for each source of variation.

Adult diet	Population	Source of variation	*df*	SSQ	*F*-ratio	*P*-value	Source of variation	*df*	SSQ	*F*-ratio	*P*-value
2.5% sugar	India	Developmental diet	2	128487.61	18.77	< 0.0001	Developmental diet	2	92118.83	13.99	< 0.0001
		Thorax length	1	17502.31	5.11	0.026	Ovariole number	1	28603.76	8.69	0.004
		Error	85	290922.44	-	-	Error	85	279820.98	-	-
	Slovakia	Developmental diet	2	21570.70	1.80	0.172	Developmental diet	2	29518.43	2.40	0.098
		Thorax length	1	13092.06	2.18	0.144	Ovariole number	1	406.52	0.07	0.798
		Error	79	473998.04	-	-	Error	79	486683.58	-	-
10% sugar	India	Developmental diet	2	66679.73	13.43	< 0.0001	Developmental diet	2	50196.87	9.56	0.0002
		Thorax length	1	17375.67	7.00	0.01	Ovariole number	1	5324.93	2.03	0.158
		Error	84	208545.47	-	-	Error	84	220596.22	-	-
	Slovakia	Developmental diet	2	29286.20	3.20	0.046	Developmental diet	2	12600.23	1.12	0.330
		Thorax length	1	89301.43	19.51	< 0.0001	Ovariole number	1	7012.69	1.25	0.267
		Error	80	366237.82	-	-	Error	80	448526.56	-	-
25% sugar	India	Developmental diet	2	29316.87	10.78	< 0.0001	Developmental diet	2	21543.05	7.60	0.001
		Thorax length	1	13619.84	10.01	0.002	Ovariole number	1	8708.94	6.14	0.015
		Error	85	115599.18	-	-	Error	85	120510.09	-	-
	Slovakia	Developmental diet	2	17824.50	3.53	0.034	Developmental diet	2	18624.32	3.24	0.044
		Thorax length	1	27736.31	10.98	0.001	Ovariole number	1	37.26	0.01	0.910
		Error	80	202170.06	-	-	Error	80	229869.11	-	-

Table S10. The results of ANOVA for the effect of population and **the yeast or sugar content** in the developmental diet, and their interactions on the fat and glycogen content at emergence. *df* - degrees of freedom; SSQ - the sum of squares for each source of variation.

Dietary component	Trait	Source of variation	*df*	SSQ	*F*-ratio	*P*-value
Yeast	Fat	Population	1	2893.82	103.93	< 0.0001
		Developmental diet	2	2059.03	36.97	< 0.0001
		Population × Developmental diet	2	6.12	0.11	0.896
		Error	30	835.36	-	-
	Glycogen	Population	1	2.10	2.17	0.151
		Developmental diet	2	7.20	3.72	0.036
		Population × Developmental diet	2	0.08	0.04	0.958
		Error	30	29.05	-	-
Sugar	Fat	Population	1	3386.42	200.08	< 0.0001
		Developmental diet	2	5166.70	152.63	< 0.0001
		Population × Developmental diet	2	79.08	2.34	0.114
		Error	30	507.76	-	-
	Glycogen	Population	1	6.97	13.26	0.001
		Developmental diet	2	17.39	16.55	< 0.0001
		Population × Developmental diet	2	6.60	6.29	0.005
		Error	30	15.76	-	-

Table S11. The results of ANOVA for the effect of population, the **yeast content** in the developmental and the adult diet, and their interactions on the fat content and glycogen content in 10-day old males. *df* - degrees of freedom; SSQ - the sum of squares for each source of variation.

Trait	Source of variation	*df*	SSQ	*F*-ratio	*P*-value
Fat	Population	1	120.13	4.08	0.046
	Developmental diet	2	3928.50	66.71	< 0.0001
	Adult diet	2	1473.78	25.03	< 0.0001
	Population × Developmental diet	2	176.63	3.00	0.055
	Population × Adult diet	2	830.92	14.11	< 0.0001
	Developmental diet × Adult diet	4	143.81	1.22	0.308
	Population × Developmental diet × Adult diet	4	383.75	3.26	0.015
	Error	90	2650.11	-	-
Glycogen	Population	1	2419.53	162.01	< 0.0001
	Developmental diet	2	15.23	0.51	0.602
	Adult diet	2	211.71	7.09	0.001
	Population × Developmental diet	2	1.73	0.06	0.944
	Population × Adult diet	2	202.22	6.77	0.002
	Developmental diet × Adult diet	4	37.00	0.62	0.650
	Population × Developmental diet × Adult diet	4	90.25	1.51	0.206
	Error	89	1329.17	-	-

Table S12. The results of ANOVA for the effect of population, the **sugar content** in the developmental and the adult diet, and their interactions on fat content and glycogen content in 10-day old males. *df* - degrees of freedom; SSQ - the sum of squares for each source of variation.

**Trait**	**Source of variation**	***df***	**SSQ**	***F*-ratio**	***P*-value**
Fat	Population	1	62.03	4.30	0.041
	Developmental diet	2	102.91	3.57	0.032
	Adult diet	2	107.45	3.73	0.028
	Population × Developmental diet	2	106.00	3.68	0.029
	Population × Adult diet	2	13.52	0.47	0.627
	Developmental diet × Adult diet	4	83.35	1.45	0.226
	Population × Developmental diet × Adult diet	4	34.73	0.60	0.662
	Error	90	1297.28	-	-
Glycogen	Population	1	1608.25	154.39	< 0.0001
	Developmental diet	2	111.67	5.36	0.006
	Adult diet	2	282.33	13.55	< 0.0001
	Population × Developmental diet	2	81.52	3.91	0.024
	Population × Adult diet	2	196.09	9.41	0.0002
	Developmental diet × Adult diet	4	95.27	2.29	0.066
	Population × Developmental diet × Adult diet	4	48.67	1.17	0.330
	Error	90	937.54	-	-

Table S13. The results of ANOVA for the effect of population, the **yeast content** in the developmental and the adult diet, and a match/mismatch between developmental and adult nutritional environment on fat content and glycogen content in 10-day old males. *df* - degrees of freedom; SSQ - the sum of squares for each source of variation.

**Trait**	**Source of variation**	***df***	**SSQ**	***F*-ratio**	***P*-value**
Fat	Population	1	120.13	2.97	0.088
	Developmental diet	2	3977.41	49.17	< 0.0001
	Adult diet	2	1524.56	18.85	< 0.0001
	Mismatch	2	140.37	1.74	0.182
	Error	100	4044.86	-	-
Glycogen	Population	1	2403.99	143.83	< 0.0001
	Developmental diet	2	16.00	0.48	0.621
	Adult diet	2	208.46	6.24	0.003
	Mismatch	2	6.20	0.19	0.831
	Error	99	1654.69	-	-

Table S14. The results of ANOVA for the effect of population, the **sugar content** in the developmental and the adult diet, and a match/mismatch between developmental and adult nutritional environment on fat content and glycogen content in 10-day old males. *df* - degrees of freedom; SSQ - the sum of squares for each source of variation.

Trait	Source of variation	*df*	SSQ	*F*-ratio	*P*-value
Fat	Population	1	62.03	4.23	0.042
	Developmental diet	2	147.86	5.05	0.008
	Adult diet	2	101.34	3.46	0.035
	Mismatch	2	69.98	2.39	0.097
	Error	100	1464.89	-	-
Glycogen	Population	1	1608.25	119.75	< 0.0001
	Developmental diet	2	109.84	4.09	0.020
	Adult diet	2	231.24	8.61	0.0004
	Mismatch	2	16.06	0.60	0.552
	Error	100	1343.03	-	-
